# Evaluation of Nutritional Status with Healthy Eating Index (HEI-2010) of Syrian Refugees Living Outside the Refugee Camps

**DOI:** 10.3390/ijerph20010849

**Published:** 2023-01-02

**Authors:** Ali Timucin Atayoglu, Yagmur Firat, Nese Kaya, Eda Basmisirli, Asli Gizem Capar, Yusuf Aykemat, Rumeysa Atayolu, Hammad Khan, Ayten Guner Atayoglu, Neriman Inanc

**Affiliations:** 1 Department of Family Medicine, International School of Medicine, Istanbul Medipol University, Istanbul 34815, Turkey; 2 Department of Nutrition and Dietetics, Faculty of Health Sciences, Erciyes University, Kayseri 38280, Turkey; 3 Department of Nutrition and Dietetics, Faculty of Health Sciences, Nuh Naci Yazgan University, Kayseri 38170, Turkey; 4 Department of Nutrition and Dietetics, School of Health Sciences, Istanbul Medipol University, Istanbul 34810, Turkey; 5 Department of Psychiatry and Behavioral Sciences, University of California, Davis, CA 95616, USA; 6 Department of Family Medicine, Beylikduzu State Hospital, Istanbul 34500, Turkey

**Keywords:** refugee, nutritional status, healthy eating index, obesity, primary health care

## Abstract

Nutrition is a public health issue. Amongst populations of refugees, unmet nutritional needs have been identified. The aim of this study was to evaluate the nutritional status of Syrian refugees living outside the refugee camps in Kayseri, Turkey. Socio-demographic data and anthropometric measurements of the refugees were collected. The relationship between diet quality, which was assessed through the Healthy Eating Index (HEI-2010), and factors (including the duration of time spent outside the refugee camp, income, obesity, and waist circumference) were measured. Four hundred refugees participated in this study. The majority of refugees (77.8%) reported a ‘poor’ diet, with the remaining filling into the ‘needs improvement’ based on HEI-2010 scores. The average consumption of fruits in the study group was 101.9 g per day (g/day), while the average consumption of vegetables was 142.2 g/day. When the relationships were examined between BMI, HEI-2010 score, the time spent as a refugee, and waist circumference, statistically significant relationships were found (*p* < 0.001). In the linear regression analysis based on these relationships, when the results were adjusted for age and gender factors, it was observed that for every year spent as a refugee, BMI score increased by 0.17 units, and waist circumference increased by 1.14 units (*p* < 0.05). As a result, this study showed that refugees have low-income-related nutritional risks. In conclusion, ensuring that refugees have access to adequate nutrient-rich food is essential; therefore, analyzing and improving nutritional standards for refugees are suggested to be part of the strategies of the public and primary health care systems.

## 1. Introduction

The refugee problem is a global issue. While it is not a new phenomenon, the size of the current refugee crisis is unprecedented. Turkey currently hosts the largest number of refugees worldwide, with an estimated 3.7 million registered Syrian refugees living in various locations across the country [1]. Moreover, in 2017, Turkey was recognized as a global leader in humanitarian assistance, spending 0.85% of its gross national income (GNI) on humanitarian causes, largely consisting of assistance to Syrian refugees residing in Turkey [2].

The 1951 Refugee Convention and 1967 Protocol are the critical legal underpinnings that established the legal status of refugees in Turkey, which were further advanced in 2016 by Turkey’s bilateral agreement with the European Council to recognize refugee status for individuals who have a fear of being persecuted due to their race, religion, nationality, and affiliation with a certain political or social group [3]. Further, these policy decisions informed massive institutional reforms within Turkey, resulting in the creation of a national asylum system, including the 2014 adoption of the Temporary Protection Regulation, which set out the legislative framework and outlined resources for those who are granted temporary protection in this country. This included the establishment of Temporary Accommodation Centers (TACs) for individuals in need of housing and other resources [1].

In this national program, free-of-charge healthcare services are provided to Syrian refugees that reside across the country, both within and outside of TACs [4,5].

Immigrants with legal status may access migrant health clinics and units for free primary healthcare services provided by the Turkish government. By August 2020, there were 2520 Syrian healthcare professionals working in these clinics and units around the country and 966 trained referral guides/translators serving patients (for linguistic problems) [6]. In addition to the more than 3.7 million Syrian refugees who are officially registered in Turkey, there are also an estimated tens of thousands to one million Syrian refugees who are not officially registered and do not have access to public health services. Furthermore, the financial strain is heavy; according to reports, Turkey has already spent over USD 30 billion on services for Syrian migrants, of which USD 10 billion went toward health care [7].

When leaving their home country and settling in their new host country, refugees typically experience a number of traumatic events, making them a vulnerable population category. Because of this, refugees are more likely to have nutritional and health inequities, which can lead to greater vulnerability to particular communicable and non-communicable diseases [8]. A study conducted in 2019 with 10,019 Syrian refugees living in Turkey found that 15.2% of participants were living with chronic illness, with diabetes, hypertension, and psychiatric disorders being the most frequently reported chronic illnesses [9]. Furthermore, according to the World Health Organization health status assessment, more than 50% of Syrian refugees in Turkey are at high risk of chronic disease, which is characterized as having three to five risk factors [10].

According to the Global Burden of Disease, one of the major determinants of chronic diseases is poor nutrition [11]. During their travels, refugees must overcome a number of nutritional challenges. They frequently have low levels of micronutrients and/or protein and caloric deficiencies when they enter the host nation. Due to the double burden of malnutrition that many regions now experience, this may be combined with a compromised starting nutritional condition in their native country. The diet and health of refugees are influenced by a variety of structural and social factors in addition to migration itself, such as their socio-economic standing, housing circumstances, cultural and linguistic difficulties, or political issues. Dietary acculturation, the process that causes immigrants to adopt the host country’s national diet, is difficult to understand [12]. Systematic reviews of acculturation among various ethnic groups in Europe have shown that the consumption of foods high in sugar, fat, and salt increases while the consumption of foods high in fruit, vegetables, whole grains, and nuts decreases [13,14]. Among other factors, such as social support, proficiency in the local language, and time and/or financial constraints, newly arrived immigrants’ diets alter over time [15]. In addition, it is stated that the risk of obesity and chronic diseases increases after immigration for those who migrate from their country for a variety of reasons, including nutritional status [16].

The Healthy Eating Index was revised and developed as HEI-2010 in 2010, according to the new diet guide of America [17]. Since our study was conducted on a group of the refugee population in central Turkey, it can be considered that there is a limitation of the HEI-2010 in the evaluation of diet quality [18]. Although there are some studies investigating diet quality in Turkey, especially using diet quality indices, it can be said that their number is insufficient [19,20,21].

The aim of this study was to evaluate the nutritional status of Syrian refugees living outside the refugee camps in Kayseri, Turkey.

## 2. Materials and Methods

### 2.1. Sample

The study was carried out between December 2017 and April 2018. Participants were identified by their residence address through the use of local registry data and referred by local professionals. The inclusion criteria were ages between 8 and 65 years old, Syrian refugee status, and living outside the TAC in Kayseri. A list was created using household identification elements, and each family received a sequence number that was further used for simple random sampling of the participants.

First of all, a preliminary study was conducted with 30 people within the scope of the study. With the preliminary results of that study, a post-hoc power analysis was carried out until the final sample number was determined as 397 (α = 0.85, *p* < 0.05).

The socio-demographic characteristics of the refugees who signed the informed consent form were determined using face-to-face interviews; additionally, nutritional status was evaluated using the HEI-2010 score, waist circumference measurements, and Body Mass Index (BMI) calculated by measuring body weight and height (kg/m^2^). The time spent as a refugee was divided into three categories as follows: ≤1 year; 1–3 years; and >3 years.

### 2.2. Data Collection

In this descriptive study, we intended to evaluate the nutritional status of the refugees who lived outside of the TACs in Kayseri. Approximately 25,000 Syrian refugees were living outside of the TACs in the city during this study; therefore, in order to obtain a 95% confidence interval with a 5% margin of error, the sample size was calculated as 379 people. As a data collection tool, a questionnaire form was developed by the researchers and translated into Arabic before being given to the participants since approximately 70% of the Syrian refugees residing in Kayseri did not speak Turkish [22]. Moreover, two Arabic translators were recruited to provide translational support in order to ensure clear communication with the subjects. The questionnaire consisted of 3 parts: (a) an evaluation of the duration of residence in Kayseri, marital and educational statuses, and income level in comparison with the data provided by the Turkish Statistical Institute (TURKSTAT)—TUIK [22]; (b) anthropometric measurements; and (c) daily food consumption behaviors. The nutritional status of the refugees was determined utilizing the validated HEI-2010 score based on a diet quality index calculation method, which had been previously created and validated by the United States Department of Agriculture’s (USDA) Center for Nutrition Policy and Promotion (CNPP) [17,23].

The HEI-2010 is a method that calculates and measures the quality of a diet in 12 components comprising nine adequacies and three moderations. These entailed total fruit, whole fruit (total fruit excluding juice), total vegetables, legumes (beans and peas), dark green vegetables, whole grains, dairy (milk, yogurt, cheese, and fortified soy beverages in the form of skim milk equivalents), total protein foods (lean fraction only), seafood, nuts and seeds, refined grains, saturated fatty acids, polyunsaturated fatty acids, monounsaturated fatty acids, sodium, calories from added sugars/solid fats/alcohol (separately), and total calories [17,18,23,24].

The HEI score for each participant was calculated using DHQ II Diet*Calc version 1.5.0 output using the SAS version 9.4 program (SAS Institute Inc., Cary NC). Per the standardized categorization methodology, if the calculated score was greater than 80, the diet quality was defined as ‘good’; a score between 51–80 evaluated the diet quality as ‘needs improvement’; and a score of less than 51 was defined as a ‘poor’ diet quality [17,18,24].

Income status was divided into two categories based on the minimum wage in Turkey at the time of this study: Lower than Minimum Wage and Higher than Minimum Wage.

### 2.3. Anthropometric Measurements

The measurements of the participant’s height, weight, and waist circumference were collected by the researchers. Height measurements were taken with a stadiometer without shoes and heels touching each other with feet 45° open (SECA Ltd., Hamburg, Germany) and the head in the Frankfort plane. Body weights were determined by sensitive standard weighing with a precision of 0.1 kg (SECA Ltd., Hamburg, Germany). BMI was calculated with the weight and height measurements of the participants (BMI = weight (kg)/height^2^ (m^2^)).

Following the WHO classification standards, participants with a BMI of less than 18.5 kg/m^2^ were classified as ‘underweight’, a BMI between 18.5–24.99 kg/m^2^ was classified as ‘normal’, and those with a BMI between 25–29.99 kg/m^2^ were classified as ‘pre-obesity’ or ‘overweight’, and those with a BMI over 30 kg/m^2^ were classified as ‘obese’ [25]. Central obesity was defined as a waist circumference greater than 80 cm in women and 94 cm in men [26]. Waist circumference measurements of the participants were performed using a non-stretch measuring tape with a precision of 0.1 cm.

Given cultural considerations, coordinators ensured that all anthropometric measurements were conducted by researchers of the same gender as the subject.

### 2.4. Ethics

The study, which was carried out between December 2017 and April 2018, started after the approval of the Institutional Ethics Committee of Erciyes University, dated 24 November 2017 and numbered 2017-527.

### 2.5. Statistical Analyses

The data were analyzed using the SPSS software (Statistical Package for Social Sciences, Statistical Software Program, Version 24.0, 2016, IBM Corp., Armonk, NY, USA). The median (IQR) for the measured variables was determined by their homogeneity. Categorical variables were expressed as numbers and percentages, and the distribution of variables was examined using a frequency table. The Chi-square test was used in the analysis of the data, the Kruskal–Wallis test was used for numerical test comparisons between groups, and the Conover test was used for post hoc examinations. A Pearson correlation test was used to examine the relationships between some data, and a linear regression analysis was also used. A *p* < 0.05 was considered statistically significant.

## 3. Results

In total, 400 Syrian refugees were included in the study, with 56.2% (*n* = 225) of the participants being male and 43.8% (*n* = 175) female, resulting in a male-female ratio of 1.28. The median age of the study group was 30 (25–42) years. The majority of participants were married (87.8%), living with their family (87.0%), and had very low incomes (73.5%). Almost half (46.3%) reported that they were unemployed or without a regular job (Table 1). When the participants were grouped and examined in terms of the time spent as refugees, it was observed that there was a statistically significant difference between the groups in terms of age, the number of individuals living together, and marriage status (*p* < 0.05) (Table 1).

The participants’ median BMI were 25.31 (22.69–27.40), while the majority of them (44.90%) were in the pre-obesity group. In addition to that, central obesity prevalence in the group was 43.20%. When the refugees were grouped in terms of their time spent as refugees, and the results of their anthropometric measurements were examined, no statistically significant difference was found between the groups (*p* > 0.05). Majority of the participants (84.0%) reported having less than three meals per day. 

The anthropometric measurements and dietary findings according to the time spent as refugees are presented in Table 2.

Table 3 demonstrates the refugees’ food consumption, food group scores, and diet quality based on the HEI-2010 score. The average consumption of fruits was 101.9 g per day (g/day), while the average consumption of vegetables was 142.2 g/day. In regard to diet quality evaluation based on the HEI-2010 scores, approximately 77.75% were categorized as ‘poor’, 22.25% as ‘needs improvement’, and none as ‘good’.

Table 4 demonstrates the relationship between the nutritional status of the refugees according to HEI-2010 and various parameters. When the participants were grouped according to the results of their HEI-2010 scores, there was no statistically significant relationship between body weight and the presence of central obesity or diet quality, while there was a significant relationship with a low-quality diet even if the income level of the participants was lower and higher than the minimum wage (*p* = 0.024, Table 4).

When the relationships between BMI, HEI-2010 score, time spent as a refugee and waist circumference were examined, statistically significant relationships were found between the time spent as a refugee and both BMI and waist circumference (*p* < 0.001). In the linear regression analysis based on these relationships, when the results were adjusted for age and gender factors, it was observed that each 1-year increase in the time spent as a refugee increased the BMI by 0.17 units and the waist circumference by 1.14 units (*p* < 0.05, Table 5 and Table 6).

## 4. Discussion

Since 2011, the Syrian Civil War has affected millions of people, with unprecedented and still growing numbers of displaced individuals taking refugee status across the globe. Following the anti-regime demonstrations that started in March 2011, the developments in Syria created one of the biggest humanitarian crises in the world. On a population basis, Syria still has the highest number of refugees or asylum-seeking populations worldwide, consisting of 12.6 million people (almost two-thirds of its population) [27]. Turkey serves millions of refugees to the host due to its strategic location (Turkey shares the longest land border with Syria). By the end of 2017, there was a 21% increase in the population of refugees in Turkey, and the largest number of asylum seekers continued to be hosted in Turkey [28]. In Turkey, the needs of refugees living in TACs are met by the Disaster and Emergency Management Authority (AFAD) by providing assistance in meeting basic needs, including housing, nutrition, health, and education [29]. Notably, past studies have identified unmet nutritional needs being a primary area of need among refugees and asylum seekers, with reports of poor outcomes, such as vitamin deficiencies, anemia, growth retardation in children, and even fatal malnutrition, amongst refugees due to inadequate nutrition [30].

In this study, we aimed to evaluate the nutritional status of Syrian refugees living outside the refugee TACs in Kayseri. For this purpose, 400 refugees aged between 18 and 65 (56.3% male and 43.8% female) were reached.

In the previous studies examining the nutrition quality and health status of the refugees, Strong et al. examined the health status of 210 elderly Syrian refugees in Lebanon and found that elderly refugees frequently skipped meals, underwent entire days without eating, or lacked essential nutrients [31]. In this study, it was seen that the income of a large portion of the refugees (83.4%) was below the minimum wage in the relevant year, as announced by TUIK. It is striking that even refugees earning a little over the minimum wage (EUR ~380) may be below the hunger limit (EUR ~450) [32]. Our study findings observed that there was no change in individuals’ income or employment status (Table 1) when the refugee groups were being shaped by their time spent as a refugee. Additionally, the number of refugee individuals living together for 1–3 or more than 3 years was statistically higher than those who have been refugees for less than 1 year (*p* = 0.001). This may also indicate an increase in the number of individuals in the family due to births, which can also cause a burden on parents (Table 1 and Table 2).

Most epidemiological studies focus on determining the relationship between a specific nutrient, food and food groups, and chronic disease risk [33,34]. However, this approach may ignore the complexities and intricacies of real-world diets. In our study, the Healthy Eating Index—one of the methodological approaches to measuring total diet quality [23]—was used. The HEI-2010 score of the group was 48 (43–51), and no participants had a ‘good’ HEI-2010 score, while 77.80% of them were in the ‘poor’ state. When the refugees in the ‘poor’ and ‘needs improvement’ groups were compared in Table 4, it was observed that there was a statistically significant difference between the groups in terms of income status (*p* = 0.024). There was no change in socio-economic status or anthropometric measurements and HEI-2010 scores between the two groups, which can indicate that they have more of a ‘quality’ rather than a ‘finding’ food problem. Based on the type of foods, relatively costlier and healthier ones have a low score of consumption by refugees distributed in Table 4 (for example, the average consumption of fruits is 101.8673 g/day, and the average consumption of vegetables is 142.2089 g/day). It can be thought that this situation is more related to income (i.e., managing everyday life).

The fact that nutrition is a highly multifactorial equation causes controversy regarding the relationship between income and nutrition and nutrition-related factors, but recently, there have been discussions of an inverse relationship between obesity and income due to the effect of income on diet quality. For example, in a systematic review/meta-analysis focusing on the relationship between income and obesity in 2017, the results of many studies, including cohort studies, were examined together and revealed that lower income is associated with subsequent obesity (OR 1.27, 95% CI 1.10 to 1.47; risk ratio 1.52, 95% CI 1.08 to 2.13), though the statistical significance vanished once adjusted for publication bias. Studies in that meta-analysis on reverse causality indicated a more consistent relationship between obesity and subsequent income, even after taking publication bias into account (standardized mean difference −0.15, 95% CI −0.30 to 0.01) [35]. In our study, it was observed that most of the Syrian refugees, most of whom have incomes below the minimum wage and hunger limit, are overweight (44.90%), and some are obese (14.60%). In addition to this information, our study results showed that there is a positive, albeit weak, relationship between the time spent during the refugee period and BMI and waist circumference (*p* < 0.001). In the linear regression analysis based on these relationships, when the results were adjusted for age and gender factors, it was observed that each 1-year increase in the time spent as a refugee increased the BMI by 0.17 units and the waist circumference by 1.14 units (*p* < 0.05) (Table 5 and Table 6). These effects of low income due to refugee status on BMI and waist circumference are consistent with the results of the systematic review/meta-analysis conducted in 2017, and based on these results, it can be thought that refugees are at risk of obesity due to their diet quality and some other related factors despite consuming few meals. In 2020, Arnotti K. and Bamber M. conducted a meta-analysis to examine the overall effects of fruit and vegetable consumption interventions on weight loss for those with a body-mass index (BMI) > 25; they followed up with moderator analyses to determine if the effects varied based on participants, interventions, methods, or source characteristics. Extensive literature searches were conducted, resulting in sixteen studies, providing seventeen comparisons across 3719 participants. The overall summary effect was large. Several moderators were significant for increased weight loss: low socio-economic status, higher baseline BMI, longer interventions, and recruitment settings (*p* < 0.05) [36]. In our study, even the fact that there was no direct correlation between HEI-2010 and anthropometric parameters, such as BMI and waist circumference, since the number of fruits and vegetable consumption is pretty low in participants, it can be hypothesized that the majority of the study group (especially in the overweight group), are at risk of obesity and that this risk may not decrease during the years spent in the country. On the other hand, it can be thought that the increase in the individual’s income and/or the given nutrition education and the increase in the consumption of vegetables and fruits (i.e., an increase in the diet quality) may cause a decrease in their overweight/obese status.

As a limitation of the study, our sampling techniques might not have highlighted the unique needs of new arrivals who may presumably be at higher risk. Additionally, the data set might have limited our ability to detect a statistically significant relationship between socio-demographic factors, such as income and HEI. Further research could include exploring the differences amongst various cities here in Turkey as well as potential parallels in refugee communities in other regions.

Our study results provide nutritional insights for a group of people who have migrated from Syria to Turkey and live in the province of Kayseri. As such, they cannot be generalized to all refugees living in Turkey or beyond. However, we think that our study results can support and contribute to the literature and cast additional light on the refugees’ plight, which needs to be further studied and analyzed in the full spectrum of social, economic, and medical conditions as well as their specific interplay. Another limitation of this study is that the food sources of the study group were not evaluated in terms of nutritional quality. It may be possible that the food available to the refugees may or may not have the quality ingredients expected.

In Turkey, when refugees go to live outside the TAC, they typically do not receive extensive assistance to successfully deal with the persistent daily problems of living in a community where they may not have the necessary language skills. We should thus point out that communication barriers can also be a limiting factor in the assessment and interpretation of nutritional status for refugees living outside the TAC.

BMI was used in the evaluation of obesity in our study. However, biometric measurements, such as BMI, are being increasingly criticized as a limited method for classifying populations since it is essentially a product of Western, white, and masculine viewpoints, which cannot truly capture complex environmental, social, and financial undercurrents at play. Further, social determinants of health are shown to be more significant in terms of understanding what are erroneously called ‘lifestyle diseases’, such as obesity.

Despite incorporating a large number of participants, our research could be improved in terms of its methodology or analysis. For instance, a more in-depth critical analysis of the possible causes of the observations, including loss of income, support, etc., could be included. The multi-variate effects of mental health and psycho-social factors that may contribute to the development of diabetes have not been addressed. Hence our findings include a particular set of nutritional data rather than a full spectrum of experiences of Syrian refugees.

## 5. Conclusions

Given the increasing trend of migration worldwide, coupled with the magnitude of existing armed conflicts, a comprehensive approach is needed to address the issues of proper nutrition among refugees. Turkey, as the first reception and transit country for many refugees due to its geographical position, focuses on humanitarian assistance, including health and nutrition. The country is making commendable efforts to provide an unprecedented influx of people with humanitarian aid and support.

The present study outlined the poor quality of nutrition among the Syrian refugees living ‘outside the TACs’ in Kayseri, Turkey. The findings presented here may provide further research directions for scientists and health management professionals.

It was shown that refugees have low-income-related nutritional risks that do not improve with their time spent as refugees. 

As one of the cornerstones of maintaining a quality level of health, nutrition is a public health issue. Therefore, public health officials are suggested to work toward creating high levels of nutrition among the general population, including refugees and immigrants. Ensuring that refugees have access to adequate nutrient-rich food is essential; therefore, analyzing and improving nutritional standards for refugees are recommended to be included in strategies of the public and primary health care systems.

The current descriptive survey offers a new and organic perspective on this topic for healthcare professionals and related experts. The refugee crisis can alter not only the social and political landscape but also impact the primary care, general health, and nutritional state of refugees for the worst in a short amount of time. 

The refugee crisis is a global issue, and there is a lot to learn from the experiences of each other. As such, it requires further and more comprehensive studies to truly unravel the many factors leading to this outcome.

## Figures and Tables

**Table 1:** Socio-demographic characteristics according to the time spent as refugees.

Variables	≤1 Year (*n* = 59)	1–3 Years (*n* = 186)	≥3 Years (*n* = 155)	*p*
	Median (IQR)	Median (IQR)	Median (IQR)	
**Age**	30 (24–34)	29 (25–39)	36 (28–45)	< 0.001 *
**Number of Individuals Living Together**	4 (3–6)	5 (4–6)	6 (5–7)	0.001 *
	***n* (%)**	***n* (%)**	***n* (%)**	
**Sex**				
Male	31 (7.75)	107 (26.75)	87 (21.75)	0.797 **
Female	28 (7.00)	79 (19.75)	68 (17.00)	
**Education**				
Primary School or Less	30 (7.50)	112 (28.00)	93 (23.25)	0.312 **
Secondary School	15 (3.75)	39 (9.75)	41 (10.25)	
High School and Above	14 (3.50)	34 (8.50)	21 (5.25)	
**Working Status**				
Employed	28 (7.00)	97 (24.20)	90 (22.50)	0.318 **
Unemployed	31 (7.80)	89 (22.20)	65 (16.20)	
**Income**				
Lower than Minimum Wage	41 (10.20)	136 (34.00)	117 (29.2)	0.666 **
Higher than Minimum Wage	18 (4.50)	44 (12.50)	35 (9.50)	
**Marital Status**				
Married	51 (12.80)	155 (38.80)	145 (36.20)	0.016 **
Single/Divorced	8 (2.20)	31 (7.80)	10 (2.50)	

* Kruskal–Wallis test (Conover test was used for post-hoc pairwise multiple comparisons procedure, *p* < 0.05); and ** Chi-Square test, *p* < 0.05.

**Table 2:** Anthropometric measurements and dietary findings according to the time spent as forced immigrants.

Variables	≤1 Year (*n* = 59)	1–3 Years (*n* = 186)	≥3 Years (*n* = 155)	*p*
	Median (IQR)	Median (IQR)	Median (IQR)	
**BMI**	25.34 (22.03–27.68)	24.97 (22.55–27.34)	25.39 (23.43–27.41)	0.381 *
**Waist Circumference**	87 (72–95)	85 (77.50–95.00)	89 (79–95)	0.626 *
**HEI-2010 Score**	49 (47–51)	48 (44–52)	46 (41–50)	**<0.001** *
	***n* (%)**	***n* (%)**	***n* (%)**	
**BMI Classification (WHO)**				
Underweight (<18.5)	5 (1.20)	9 (2.20)	6 (1.5)	0.576 **
Normal weight (18.5–24.9)	23 (5.8)	85 (21.20)	59 (14.80)	
Pre-obesity (overweight) (25–29.9)	25 (6.20)	78 (19.50)	77 (19.20)	
Obesity (≥30)	6 (1.50)	14 (3.50)	13 (3.20)	
**Central Obesity Presence**				
Yes	28 (7.00)	76 (19.00)	69 (17.20)	0.619 **
No	31 (7.80)	110 (27.50)	86 (21.50)	
**Number of Meals Per Day**				
<3	47 (11.80)	151 (37.80)	138 (34.50)	0.089 **
3–6	12 (3.00	35 (8.80)	17 (4.20)	
**Skipping Meals**				
No	21 (5.20)	82 (20.50)	77 (19.20)	0.184 **
Skipping Breakfast	17 (4.20)	35 (8.80)	24 (6.00)	
Skipping Lunch or Dinner	21 (5.20)	69 (17.20)	54 (13.50)	

* Kruskal–Wallis test (Conover test was used for post-hoc pairwise multiple comparisons procedure, *p <* 0.05); and ** Chi-Square test, *p* < 0.05.

**Table 3:** Refugees’ food consumption, food group scores, and diet quality, as based on their HEI-2010 scores.

**Food Type**	**Mean (SD)**
Total Fruit ^1^ (g)	101.87 (78.47)
Whole Fruit ^2^ (g)	96.29 (70.47)
Total Vegetables ^3^ (g)	142.21 (75.83)
Greens and Beans ^3^ (g)	27.24 (15.04)
Whole Grains (g)	3.32 (13.57)
Dairy ^4^ (g)	166.24 (87.91)
Total Protein Foods ^5^ (g)	34.57 (23.81)
Seafood and Plant Proteins ^5,6^ (g)	32.87 (6.05)
Fatty Acids ^7^ (g)	1.38 (0.40)
Refined Grains (g)	162.87 (67.64)
Sodium (mg)	1038.63 (554.63)
Empty Calories ^8^ (%)	2.38 (0.72)
**Food Scores**	**Mean (SD)**
Total Fruit ^1^ (g)	2.26 (1.68)
Whole Fruit ^2^ (g)	3.34 (1.96)
Total Vegetables ^3^ (g)	3.73 (1.54)
Greens and Beans ^3^ (g)	2.43 (0.84)
Whole Grains (g)	0.39 (1.30)
Dairy ^4^ (g)	4.84 (2.68)
Total Protein Foods ^5^ (g)	1.90 (0.52)
Seafood and Plant Proteins ^5,6^ (g)	4.15 (0.55)
Fatty Acids ^7^ (g)	2.03 (2.47)
Refined Grains (g)	1.98 (2.75)
Sodium (mg)	0 (0)
Empty Calories ^8^ (%)	20.0 (0)
Total	47.04 (5.88)
**Diet Quality Based on the HEI-2010 Scores**	***n* (%)**
Poor	311 (77.75)
Needs improvement	89 (22.25)
Good	0

^1^: Includes 100% fruit juice. ^2^: Includes all forms except juice. ^3^: Includes any beans and peas not counted as Total Protein Foods. ^4^: Includes all milk products, such as fluid milk, yogurt, cheese, and fortified soy beverages. ^5^: Beans and peas are included here (and not with vegetables) when the Total Protein Foods standard is not otherwise met. ^6^: Includes seafood, nuts, seeds, soy products (other than beverages), as well as beans and peas, which are counted as Total Protein Foods. ^7^: Ratio of poly- and mono-unsaturated fatty acids (PUFAs and MUFAs) to saturated fatty acids (SFAs). ^8^: Calories from solid fats, alcohol, and added sugars (the threshold for counting alcohol is >13 g/1000 kcal) [17,18,24].

**Table 4:** Relationship between the nutritional status of the refugees according to HEI-2010 and various parameters.

Variables	Poor Diet (*n* = 311)	Needs Improvement (*n* = 89)	*p*
	*n* (%)	*n* (%)	
**Time spent as a refugee**			
≤1 Year	111 (35.7)	32 (35.90)	0.104
1–3 Years	174 (55.90)	55 (61.80)	
≥3 Years	26 (8.40)	2 (2.30)	
**Income, *n* (%)**			
Lower than Minimum Wage	221 (71.10)	73 (82.00)	0.024 *
Higher than Minimum Wage	90 (22.50)	16 (4.00)	
**BMI (kg/m^2^), *n* (%)**			
Underweight (<18.5)	17 (5.50)	3 (3.40)	0.410
Normal weight (18.5–24.9)	124 (39.90)	43 (48.30)	
Pre-obesity (25–29.9)	142 (45.60)	38 (42.70)	
Obesity (≥30)	28 (9.00)	5 (5.60)	
**Central Obesity, *n* (%)**			
Yes	180 (57.90)	47 (52.80)	0.395
No	131 (42.10)	42 (47.20)	

* Chi-Square Test, *p* < 0.05.

**Table 5:** The relationship between BMI, HEI-2010 score, waist circumference, and time spent as a refugee.

Variables	Time Spent as A Refugee	
	*r*	*p*-Value
BMI	0.161	<0.001 *
HEI-2010	−0.077	0.122
Waist Circumference	0.169	<0.001 *

* Pearson correlation test, *p* < 0.05.

**Table 6:** The relationship between time spent as a refugee, BMI, and waist circumference.

Models		BMI				Waist Circumference			
		Coefficient (B)	95% Confidence Interval		*p*-Value	Coefficient (B)	95% Confidence Interval		*p*-Value
			Lower	Upper			Lower	Upper	
Time spent as a refugee	Model 0	0.22	23.94	25.09	<0.001	1.02	83.95	87.36	<0.001
	Model 1	0.17	21.17	23.64	0.006	1.14	79.11	89.69	<0.001

Model 0: No adjustments made for independent variables that are thought to be confounding, Model 1: Adjusted for age and gender (the potential correlations of confounding factors and their possible multicollinearity were examined between the parameters); *p* < 0.05.

## Data Availability

Data will be available from the corresponding author upon reasonable request.
